# A large-scale study on the prevalence of intestinal parasites in patients referred to medical laboratories in Urmia, Northwest Iran

**DOI:** 10.1186/s12876-023-02947-5

**Published:** 2023-09-20

**Authors:** Shiva Zeinali, Mahsa Rezgi, Morteza Gholinejad, Rasool Jafari

**Affiliations:** 1 Student Research Committee, Urmia University of Medical Sciences, Urmia, Iran; 2 Department of Parasitology and Mycology, School of Medicine, Urmia University of Medical Sciences, Urmia, Iran; 3 Cellular and Molecular Research Center, Cellular and Molecular Medicine Research Institute, Urmia University of Medical Sciences, Urmia, Iran

**Keywords:** Intestinal parasites, Urmia, Iran

## Abstract

**Introduction:**

Intestinal parasitic infections (IPIs), caused by helminths and protozoans, are among the most prevalent infections in humans in developing countries. This study aimed to determine the prevalence of IPIs in patients referred to three educational and medical centers affiliated with Urmia University of Medical Sciences in Urmia.

**Materials and methods:**

In this cross-sectional study, 2845 stool samples, including 2174 (76.4%) males and 671 (23.6%) females, were collected from patients referred to Imam Khomeini and Shahid Motahhari hospitals and Shahid Nikkhah Health Center in Urmia, Northwest Iran, from January 2020 to February 2022. The microscopic examination for IPIs was carried out using the wet mount method, and the hard-to-identify samples were stained by trichrome for accurate identification of protozoa. For diagnosis of infections by coccidian parasites modified Ziehl-Neelsen (mZN) staining was used.

**Results:**

Based on the results, two hundred nine intestinal parasites were identified in 184 out of 2845 (6.5%) patients of which 136 out of 2174 males (6.3%) and 48 out of 671 females (7.2%) were positive. Some patients had tested positive for multiple protozoa. The observed intestinal protozoa are as follows: *Blastocystis spp.* 118 (4.1%), *Endolimax Nana* 42 (1.5%), *Entamoeba coli* 24 (0.8%), *Giardia lamblia* 13 (0.5%), *Cryptosporidium* spp. 6 (0.2%), *Iodamoeba butschlii* 3 (0.1%), *Chilomastix mesnili* 2 (0.1%), and an accidentally detected helminthic infection *Enterobius vermicularis* 1 (0.05%).

**Conclusion:**

According to the results, the most prevalent IPIs in West Azerbaijan Province are caused by *Blastocystis* spp., and *Giardia lamblia*. Most intestinal protozoa observed in the study were nonpathogenic and commensal, which shows water or food contamination in the area. Thus, medical technologists in the parasitology section must be trained and aware of IPIs in medical laboratories.

## Introduction

Intestinal parasite infections are a major public health concern, especially in low- and middle-income countries [1]. Intestinal parasitic infections (IPIs) morbidities differ among individuals, depending on parasite factors such as the type, load, and intensity of intestinal parasites [2–4] and host factors especially age and nutritional status [4]. Intestinal parasites are common among school-aged children [2–5]. Lack of soap for handwashing [4], safe water, and sanitation are thought to be important risk factors for acquiring IPIs [2].

Intestinal parasites are abundant and infect millions of people all around the globe in the tropics and developing countries. However the potential transmission of intestinal parasites in developed countries and temperate zones has recently become problematic, especially among immigrants, returning travelers, and humans with immune system disorders [1].

Currently, on a global scale, age-specific mortality has gradually improved over the past 35 years; this pattern of overall development has been sustained in the past decade [6]. IPIs also declined dramatically during recent decades, mostly because of increased hygienic status, health education, and sanitation [7], even in African developing countries the decline in parasitic infections is noticeable [8].

One of the very important complications regarding intestinal parasites, especially intestinal helminths, is the nutritional effect, and growth retardation in children. These effects depend on the species of parasite, coinfection with multiple parasites, the duration of infection, and the load of worms. The influence of infections on different individuals also depends on the nutritional status of the host. Treating helminthic infections can lead to improvements in nutritional status and consequently in growth [2]. Based on what was discussed formerly, the diagnosis of IPIs is crucial and it is prerequired for treatment. On the other hand, because of the declining trend of most parasitic infections, having new updates on the prevalence of these infections in every region will help physicians and healthcare authorities to be aware of the situation in their region. Thus, the present study aimed to determine the prevalence of IPIs in a large group of patients referred to three healthcare facilities in Urmia, Northwest Iran.

## Materials and methods

### Samples collection

In this cross-sectional study, 2845 stool samples were collected from patients referred from different regions of West Azerbaijan Province to Imam Khomeini and Shahid Motahhari hospitals and Shahid Nikkhah Health Center in Urmia, Northwest Iran, from January 2020 to February 2022. Patients were informed about the study and a questionnaire of demographic variables was cpmpleted by interviewing every patient. The collected stool samples were transferred to the parasitology laboratory in the Department of Parasitology and Mycology, School of Medicine, Urmia University of Medical Sciences, Urmia, Iran, for further laboratory workouts.

### COVID-19 pandemic

Two months after the initiation of sampling, COVID-19 quarantine began in Iran, thus 649 samples were collected before the COVID-19 pandemic quarantine and 2196 samples were collected during the pandemic quarantine.

### Samples preparation

Stool samples were divided into two parts in a suitable stool container, one preserved by 4% formalin to be used for wet mount and modified cold Ziehl–Neelsen staining (mZN), and the other preserved in polyvinyl alcohol (PVA) to be used for trichrome staining when needed [9].

### Microscopic examination

Samples were examined microscopically by the wet mount method with normal saline or Lugol’s iodine stain using 10X and 40X objectives. The identification was solely based on the morphological features of the parasites and those with unidentifiable organisms by the wet mount, were then stained with the trichrome staining method and investigated under the 100X objective [10]. For the diagnosis of coccidian parasites, mZN was used and studied with the 100X objective [11].

### Limitations of the study

Stool samples were studied by the direct wet mount, trichrome, and mZN staining methods without formalin-ethyl-acetate concentration, and other species-specific methods, such as adhesive cellophane tape for detecting *Enterobius vermicularis* eggs or agar plate culture for *Strongyloides stercoralis*, could not be performed. Thus, we studied and reported the prevalence of intestinal infections/colonizations that the parasite could be detected by wet mount, trichrome staining, and mZN on stool specimens.

### Data analyses

Data of findings from the microscopic examination and variables in the questionnaires were analyzed by IBM SPSS Statistics for Windows, version 23 (IBM Corp., Armonk, N.Y., USA) using chi-square, Mann-Whitney, *t*-, and logistic regression tests depending on the variable tested. Throughout the manuscript, we used infection when referring to a pathogenic parasite, colonization when referring to a nonpathogenic organism, and infection/colonization when referring to pathogenic and nonpathogenic organisms together.

## Results

Over two years, 2845 stool samples were collected, including 2174 (76.4%) males and 671 (23.6%) females with an age range of 1–87 years and a mean age of 32.86 years. Stool examination (wet mount with saline and Lugol’s iodine, trichrome, and mZN) resulted in 209 infections/colonizations in 184 out of 2845 humans (6.5%), among whom 136 out of 2174 males (6.3%), and 48 out of 671 females (7.2%) were positive (*p* = 0.409).

Total IPIs/C was more prevalent in patients aged 40–49 and 50–59 years than in patients aged 0–9 years, with odds ratios of 2.369 (*p* = 0.021) and 3.218 (*p* = 0.002), respectively. The seasonal prevalence showed the highest rates in winter, and the prevalence was significantly lower in summer and fall than in winter, with the odds ratio of 0.528 and 0.564, respectively (*P* = 0.02). The prevalence of IPIs/Cs was higher in diarrheic patients than in those with normal stool (OR = 3.613; *P* = 0.002) (Table 1).

**Table 1:** Odds ratios estimated for total IPIs/Cs among sexes, age groups, habitat, season, COVID-19 pandemic period, and stool consistency. The odds ratio is calculated for being positive

**Variables**		Total infection/colonization		Total	Odds ratio	*P* value
		Negative	Positive			
Sex n (%)	Male	2038 (93.7)	136 (6.3)	2174(100)	0.896	0.409
	Female	623 (92.8)	48 (7.2)	671 (100)	1	
	Total	2661 (93.5)	184 (6.5)	2845 (100)		
Age groups* n (%)	0–9	254 (96.2)	10 (3.8)	264(100)	1	Contrast var.
	10–19	331 (93.8)	22 (6.2)	353(100)	1.789	0.143
	20–29	670 (94.6)	38 (5.4)	708(100)	1.586	0.209
	30–39	605 (93.8)	40 (6.2)	645(100)	1.867	0.089
	40–49	425 (92.0)	37(8.0)	462(100)	2.369	0.021
	50–59	273 (89.5)	32 (10.5)	305 (100)	3.218	0.002
	60–69	81 (94.2)	5(5.8)	86(100)	1.684	0.359
	70–79	19 (100)	0 (0)	19(100)	-	-
	> 80	3 (100)	0 (0)	3(100)	-	-
	Total	2661 (93.5)	184(6.5)	2845 (100)		
Habitat n (%)	Urban	2015 (93.3)	144(6.7)	2159(100)	1.154	0.436
	Rural	646 (94.2)	40(5.8)	686(100)	1	
	Total	2661 (93.5)	184(6.5)	2845(100)		
Season* n (%)	Spring	105 (96.3)	4 (3.7)	109(100)	0.373	0.063
	Summer	860 (94.3)	52 (5.7)	912(100)	0.528	0.02
	Fall	1100 (94.5)	64 (5.5)	1164(100)	0.564	0.02
	Winter	596 (90.3)	64 (9.7)	660(100)	1	Contrast var.
	Total	2661 (93.5)	184 (6.5)	2845(100)		
Covid n (%)	Pre-covid	616 (94.9)	33 (5.1)	649(100)	0.697	0.103
	Post-covid	2045 (93.1)	151 (6.9)	2196(100)	1	
	Total	2661 (93.5)	184 (6.5)	2845(100)		
Stool consistency* n (%)	Formed	756 (94.1)	47 (5.9)	803 (100)	1	Contrast var.
	Soft	1196 (93.7)	80 (6.3)	1276 (100)	1.100	0.618
	Loose	663 (93.1)	49 (6.9)	712 (100)	1.216	0.361
	Watery	46 (85.2)	8 (14.8)	54 (100)	3.613	0.002
	Total	2661 (93.5)	184 (6.5)	2845 (100)		

* Odds ratio of the variables with more than two variants was estimated by binary logistic regression test

The observed intestinal protozoa in order from highest to lowest frequency are as follows: *Blastocystis* spp. 118 (4.1%), *Endolimax nana* 42 (1.5%), *Entamoeba coli* 24 (0.8%), *Giardia lamblia* 13 (0.5%), *Cryptosporidium* spp. 6 (0.2%), *Iodamoeba butschlii* 3 (0.1%), *Chilomastix mesnili* 2 (0.1%), and an accidentally detected helminth *Enterobius vermicularis* 1 (0.05%). No other parasitic elements other than the mentioned ones were detected (Table 2).

**Table 2:** Odds ratio estimated for being positive in different observed intestinal protozoa among sexes, age groups, habitats, and seasons. The chi-square test was performed on 2 × 2 tables yet for the variables with more than two variants, a binary logistic regression test was used to estimate the *P* value

			Sex		Age groups									Habitat		Season			
**Parasite**		Total	Male	Female	0–9	10–19	20–29	30–39	40–49	50–59	60–69	70–79	> 80	Urban	Rural	Spring	Summer	Fall	Winter
*E. nana* n (%)	Negative	2803 (98.5)	2142 (98.5)	661 (98.5)	261 (98.9)	349 (98.9)	703 (99.3)	633 (98.1)	452 (97.8)	297 (97.4)	86 (100)	19 (100)	3 (100)	2126 (98.5)	677 (98.7)	109 (100)	898 (98.5)	1152 (99.0)	644 (97.6)
	Positive	42 (1.5)	32 (1.5)	10 (1.5)	3 (1.1)	4 (1.1)	5 (0.7)	12 (1.9)	10 (2.2)	8 (2.6)	0 (0)	0 (0)	0 (0)	33 (1.5)	9 (1.3)	0 (0)	14 (1.5)	12 (1.0)	16 (2.4)
	Total	2845	2174	671	264	353	708	645	462	305	86	19	3	12,159	686	109	912	1164	660
	OR		0.971	1	1	0.929	0.605	1.603	1.734	2.149	-	-	-	1.168	1	-	0.635	0.448	1
	*P*		0.972		*	0.924	0.495	0.47	0.41	0.266	-	-	-	0.682		-	0.222	0.039	*
*E. coli* n (%)	Negative	2821 (99.2)	2159 (99.3)	662 (98.7)	262 (99.2)	351 (99.4)	707 (99.9)	638 (98.9)	457 (98.9)	298 (97.7)	86 (100)	19 (100)	3 (100)	2137 (99.0)	684 (99.7)	109 (100)	902 (98.9)	1155 (99.2)	655 (99.2)
	Positive	24 (0.8)	15 (0.7)	9 (1.3)	2 (0.8)	2 (0.6)	1 (0.1)	7 (1.1)	5 (1.1)	7 (2.3)	0 (0)	0 (0)	0 (0)	22 (1.0)	2 (0.3)	0 (0)	10 (1.1)	9 (0.8)	5 (0.8)
	Total	2845	2174	671	264	353	708	645	462	305	86	19	3	2159	686	109	912	1164	660
	OR		0.510	1	1	0.749	0.181	1.419	1.398	2.989	-	-	-	3.521	1	-	1.491	1.39	1
	*P*		0.107		*	0.775	0.164	0.666	0.693	0.179	-	-	-	0.070		-	0.470	0,829	*
*Blastocystis* spp. n (%)	Negative	2727 (95.9)	2087 (96)	640 (95.4)	261 (98.9)	337 (95.5)	680 (96.0)	619 (96.0)	438 (94.8)	288 (94.4)	82 (95.3)	19 (100)	3 (100)	2070 (95.9)	657 (95.8)	105 (96.3)	888 (97.4)	1118 (96.0)	616 (93.3)
	Positive	118 (4.1)	87 (4.0)	31 (4.6)	3 (1.1)	16 (4.5)	28 (4.0)	26 (4.0)	24 (5.2)	17 (5.6)	4 (4.7)	0 (0)	0 (0)	89 (4.1)	29 (4.2)	4 (3.7)	24 (2.6)	46 (4.0)	44 (6.7)
	Total	2845	2174)	671	26	353	708	645	462	305	86	19	3	2159	686	109	912	1164	660
	OR		0.86	1	1	4.319	3.804	3.909	4.893	5.365	4.718	-	-	0.973	1	0.518	0.368	0.614	1
	*P*		0.483		*	0.022	0.029	0.027	0.01	0.008	0.046	-	-	0.904		0.218	< 0.001	0.026	*
*G. lamblia* n (%)	Negative	2832 (99.5)	2162 (99.4)	670 (99.9)	262 (99.2)	352 (99.7)	705 (99.6)	643 (99.7)	459 (99.4)	304 (99.7)	85 (98.8)	19 (100)	3 (100)	2148 (99.5)	684 (99.7)	109 (100)	904 (99.1)	1164 (100)	655 (99.2)
	Positive	13 (0.5)	12 (0.6)	1 (0.1)	2 (0.8)	1 (0.3)	3 (0.4)	2 (0.3)	3 (0.6)	1 (0.3)	1 (1.2)	0 (0)	0 (0)	11 (0.5)	2 (0.3)	0 (0)	8 (0.9)	0 (0)	5 (0.8)
	Total	2845	2174	671	264	353	708	645	462	305	86	19	3	2159	686	109	912	1164	660
	OR		0.27	1	-	-	-	-	-	-	-	-	-	0.57	1	-	-	-	-
	*P*		0.176		0.956									0.461		0.013			
*I. butschlii* n (%)	Negative	2842 (99.9)	2171 (100)	671 (100)	264 (100)	353 (100)	707 (99.9)	645 (100)	460 (99.6)	305 (100)	86 (100)	19 (100)	3 (100)	2156 (99.9)	686 (100)	109 (100)	911 (99.9)	1162 (99.8)	660 (100)
	Positive	3 (0.1)	3 (0.1)	0 (0)	0 (0)	1 (0.3)	0 (0)	0 (0)	2 (0.4)	0 (0)	0 (0)	0 (0)	0 (0)	3 (0.1)	0 (0)	0 (0)	1 (0.1)	2 (0.2)	0 (0)
	Total	2845	2174	671	264	353	708	645	462	305	86	19	3	2159	686	109	912	1164	660
	OR		0.76	1	-	-	-	-	-	-	-	-	-	0.76	1				
	P		0.33		0.585									0.329		0.729			
* C. mesnili* n (%)	Negative	2843 (99.9)	2173 (99.95)	670 (99.9)	264 (100)	352 (99.7)	708 (100)	645 (100)	462 (100)	304 (99.7)	86 (100)	19 (100)	3 (100)	2157 (99.9)	686(100.0)	109 (100)	911 (99.9)	1164 (100)	659 (99.8)
	Positive	2 (0.1)	1 (0.046)	1 (0.1)	0 (0)	1 (0.3)	0 (0)	0 (0)	0 (0)	1 (0.3)	0 (0)	0 (0)	0 (0)	2 (0.1)	0 (0)	0 (0)	1 (0.1)	0 (0)	1 (0.2)
	Total	2845	2174	671	264	353	708	645	462	305	86	19	3	2159	686	109	912	1164	660
	OR		0.308	1	-	-	-	-	-	-	-	-	-	1.333	1	-	-	-	-
	*P*		0.379		0.569									0.425		0.633			
*Cryptosporidium* n (%)	Negative	2839 (99.8)	2169 (99.8)	670 (99.9)	262 (99.2)	353 (100)	707 (99.9)	644 (99.8)	460 (99.6)	305 (100)	86 (100)	19 (100)	3 (100)	2155 (99.8)	684 (99.7)	109 (100)	910 (99.8)	1161 (99.7)	659 (99.8)
	Positive	6 (0.2)	5 (0.2)	1 (0.1)	2 (0.8)	0 (0)	1 (0.1)	1 (0.2)	0 (0.4)	0 (0)	0 (0)	0 (0)	0 (0)	4 (0.2)	2 (0.3)	0 (0)	2 (0.2)	3 (0.3)	1 (0.2)
	Total	2845	2174	671	264	353	708	645	462	305	86	19	3	2159	686	109	912	1164	660
	OR		1.545	1	1	-	0.183	0.239	0.605	-	-	-	-	1.575	1	-	2.174	1.446	1
	*P*		0.568		*	-	0.172	0.252	0.630	-	-	-	-	0.597		-	0.530	0.744	*

* Contrast variable

Cryptosporidiosis was observed only in patients with diarrhea and loose stool. No statistically significant difference was observed solely for other observed protozoa with the consistency of stool specimens (Table 3).

**Table 3:** Odds ratio estimated for stool consistency among different IPIs/Cs using binary logistic regression test

			Consistency			
Parasite		Total	Formed	Soft	Loose	Watery
*E. nana*	Negative n (%)	2803(98.5)	789(98.3)	1259(98.7)	703(98.7)	52 (96.3)
	Positive n (%)	42(1.5)	14(1.7)	17(1.3)	9(1.3)	2 (3.7)
	Total	2845(100)	803(100)	1276(100)	712(100)	54 (100)
	Odds ratio		1	0.761	0.721	2.168
	P value		Contrast var.	0.453	0.448	0.315
*E. coli*	Negative n (%)	2821(99.2)	798(99.4)	1263(99.0)	707(99.3)	53(98.1)
	Positive n (%)	24(0.8)	5(0.6)	13(1.0)	5(0.7)	1(1.9)
	Total	2845(100)	803(100)	1276(100)	712(100)	54(100)
	Odds ratio		1	1.643	1.129	3.011
	P value		Contrast var.	0.347	0.849	0.318
*Blastocystis* spp.	Negative n (%)	2727(95.9)	776(96.6)	1223(95.8)	676(94.9)	52(96.3)
	Positive n (%)	118(4.1)	27(3.4)	53(4.2)	36(5.1)	2(3.7)
	Total	2845(100)	803(100)	1276(100)	712(100)	54(100)
	Odds ratio		1	1.246	1.531	1.105
	P value		Contrast var.	0.362	0.102	0.893
*G. lamblia*	Negative n (%)	2832(99.5)	798(99.4)	1270(99.5)	710(99.7)	549(100)
	Positive n (%)	13(0.5)	5(0.6)	6(0.5)	2(0.3)	0(0)
	Total	2845(100)	803(100)	1276(100)	712(100)	54(100)
	Odds ratio		1	0.754	0.450	-
	P value		Contrast var.	0.642	0.340	-
*I. butschlii*	Negative n (%)	2842(99.9)	803(100)	1273(99.8)	712(100)	54(100)
	Positive n (%)	3(0.1)	0(0)	3(0.2)	0(0)	0(0)
	Total	2845(100)	803(100)	1276(100)	712(100)	54(100)
	Odds ratio		-	-	-	-
	P value		-	-	-	-
*C. mesnili*	Negative n (%)	2843(99.9)	802(99.9)	1276(100)	712(100)	53(98.1)
	Positive n (%)	2(0.1)	1(0.1)	0 (0)	0(0)	1(1.9)
	Total	2845(100)	803(100)	1276(100)	712(100)	54(100)
	Odds ratio		-	-	-	-
	P value		-	-	-	-
*Cryptosporidium* spp.	Negative n (%)	2839(99.8)	803(100)	1276(100)	709 (99.6)	51(94.4)
	Positive n (%)	6(0.2)	0(0)	0(0)	3(0.4%)	3(5.6%)
	Total	2845(100)	803(100)	1276(100)	712(100)	54(100)
	Odds ratio		-	-	0.072	1
	P value		0.99	0.986	0.02	Contrast var.

A fraction of samples were collected before the quarantine during the COVID-19 pandemic, 649 out of 2845 samples (22.8%). The prevalence of intestinal protozoa was not significantly different in the pre- and post-COVID-19 periods, yet *E. nana* was more prevalent in the post-COVID-19 period than in the pre-COVID-19 period (OR = 16.66; *P* = 0.005) (Table 4).

**Table 4:** Odds ratios and *P* values estimated for different IPIs/Cs in the pre- and post-COVID-19 periods. The odds ratios were calculated for being negative for each infection and can be inversed by 1 ÷ OR

Parasite		Covid pandemic		Total
		Pre-covid	Post-covid	
Total IPIs/C	Negative n (%)	616 (23.1)	33 (17.9)	649 (22.8)
	Positive n (%)	2045 (76.9)	151 (82.1)	2196 (77.2)
	Total	2661 (100)	184 (100)	2845 (100)
	Odds ratio	1.378	1	
	*P* value	0.1		
*E. nana*	Negative n (%)	647 (99.7)	2156 (98.2)	2803 (98.5)
	Positive n (%)	2 (0.3)	40 (1.8)	42 (1.5)
	Total	649 (100)	2196 (100)	2845 (100)
	Odds ratio	6.002	1	
	P value	0.005		
*E. coli*	Negative n (%)	649 (100)	2172 (98.9)	2821 (99.2)
	Positive n (%)	0 (0)	24 (1.1)	24 (0.8)
	Total	649 (100)	2196 (100)	2845 (100)
	Odds ratio	-	-	
	P value	0.007		
*Blastocystis*	Negative n (%)	621 (95.7)	2106 (95.9)	2727 (95.9)
	Positive n (%)	28 (4.3)	90 (4.1)	118 (4.1)
	Total	649 (100)	2196 (100)	2845 (100)
	Odds ratio	0.948	1	
	P value	0.808		
*G. lamblia*	Negative n (%)	645 (99.4)	2187 (99.6)	2832 (99.5)
	Positive n (%)	4 (0.6)	9 (0.4)	13 (0.5)
	Total	649 (100)	2196 (100)	2845 (100)
	Odds ratio	0.664	1	
	P value	0.493		
*I. butschlii*	Negative n (%)	649 (100)	2193 (99.9)	2842 (99.9)
	Positive n (%)	0 (0)	3 (0.1)	3 (0.1)
	Total	649 (100)	2196 (100)	2845 (100)
	Odds ratio	-	-	
	P value	0.346		
* C. mesnili*	Negative n (%)	649 (100)	2194 (99.9)	2843 (99.9)
	Positive n (%)	0 (0)	2 (0.1)	2 (0.1)
	Total	649 (100)	2196 (100)	2845 (100)
	Odds ratio	-	-	
	P value	0.442		
*Cryptosporidium*	Negative n (%)	649 (100)	2190 (99.7)	2839 (99.8)
	Positive n (%)	0 (0)	6 (0.3)	6 (0.2)
	Total	649 (100)	2196 (100)	2845 (100)
	Odds ratio	-	-	
	P value	0.183		

Considering coinfections, 21 out of 184 patients (11.41%) had mixed infections/colonizations with two (81%) or three (19%) protozoa. There was no patient coinfected by *Cryptosporidium* with other parasites and all six patients had a single infection. The most frequent coinfections/colonizations in order from the highest to lowest were as follows: *E. coli*-*Blastocystis* spp., *Blastocystis* spp.-*G. lamblia*, *E. nana*-*E. coli*, *E. nana*-*E. coli*-*Blastocystis* spp., and *E. nana*-*Blastocystis* spp. (Table 5).

**Table 5:** The observed mixed infections among the 2845 studied humans

Frequency	*E. nana*	*E. coli*	*Blastocystis* spp.	*G. lamblia*	*I. butschlii*	*C. mesnili*	*E. vermicularis*	Mixed Infection
3 (14.3%)			*	*				Double
1 (4.75%)			*				*	Double
1 (4.75%)			*		*			Double
3 (14.3%)	*	*						Double
1 (4.75%)	*			*				Double
1 (4.75%)	*		*		*			Triple
4 (19.05%)		*	*					Double
3 (14.3%)	*	*	*					Triple
3 (14.3%)	*		*					Double
1 (4.75%)	*					*		Double
Total: 21								

The mean age of the patients with IPIs/C (35.75 years) was significantly higher (*p* = 0.005) than that of the noninfected patients (32.67 years) when tested by a nonparametric Mann-Whitney test.

## Discussion

In the present study, a considerable fraction (6.5%) of humans referred to health care facilities of Urmia University of Medical Sciences, in Urmia had IPIs/Cs, however; some of the observed organisms were pathogenic, and some were nonpathogenic. The patients were referred from different regions of West Azerbaijan Province. The observed IPIs/Cs in order from highest to lowest were *Blastocystis spp.*, *Endolimax nana*, *Entamoeba coli*, *Giardia lamblia*, *Cryptosporidium* spp., *Iodamoeba butschlii*, *Chilomastix mesnili*, and *Enterobius vermicularis*. As is obvious, intestinal helminthic infections were not observed except for one accidental case of *E. vermicularis* in stool, which is regularly analyzed either from adhesive cellophane tape or swab samples [12] that were not performed in this study. All other observed ones were protozoa, which are mostly transmitted by the fecal-oral route. Some of the observed infections are known to be associated with diarrhea such as giardiasis and cryptosporidiosis [13]. Other important parasites, such as *Entamoeba histolytica*, intestinal soil-transmitted helminths, intestinal tapeworms, and hepatic and intestinal trematodes were not observed in the patients using stool examination, which is very promising because in past decades, intestinal parasitic infections were highly prevalent in Iran [14–16] and in Urmia [17, 18]. However; in the present study direct stool samples were not concentrated by any method. Considering that the concentration method increases the sensitivity of detecting some parasitic infections, there may be a probability that some IPIs were underreported by the wet mount technique [19].

Hazrati-Tapeh et al. (2010) studied the prevalence of intestinal parasitic infections among mentally disabled children and adults in Urmia, the same region as the present study, but in a different population and by a more sensitive formalin-ether concentration method. They reported the overall prevalence of the infection as 20.4% among which 17.3% had protozoal infection and 3.1% had *Enterobius vermicularis* eggs. Their reported protozoa were as follows: *Entamoeba coli* 9.7%, *Giardia lamblia* 6.2%, *Iodamoeba butschlii* 5.7%, *Blastocystis* 4%, and *Entamoeba histolytica/dispar* 0.4%. Their reported protozoa are also reported in the present study, except *E. histolytica*/*dispar* [17]. However, their reported prevalences in most of the parasites (except *Blastocystis*) are considerably higher than in the present study with different studied populations. This dramatic decline in the prevalence of intestinal parasites is likely the result of the recent improvement in hygienic status, sanitation, and health education in society. The recent decline in the prevalence of intestinal parasites has also occurred in other parts of the country that parasitic infections were prevalent in the past [14–18]. This reduction is also true for *Cryptosporidium* spp. infection, as Nuri et al. (1991) reported 7.66% in patients with diarrhea [18], and Hazrati-Tapeh et al. (1970) reported 11.5% in renal transplant recipients and 3.88% in hemodialysis patients [20], which are noticeably higher than our findings, however in a different studied population.

*Blastocystis* is classified in phylum stramenopile and is the most common enteric protozoa in the human intestinal tract [21]. Currently, it is genetically classified into at least 34 subtypes (STs) [22]. ST1–9 and 12 are reported in humans and more than 90% of human infections are caused by ST1–4 [21]. Despite the high prevalence, the public health significance and pathogenicity remain uncertain. *Blastocystis* has been reported frequently in healthy individuals. It is also reported to decrease the population of gut microbiota e.g. *Bifidobacterium* and *Lactobacillus* [21, 23]. The zoonotic aspect of *Blastocystis* is also hypothesized and may contribute to some infections [21]. *Blastocystis* is also reported to be a neglected cause of urticaria and some skin disorders and is suggested to be treated in patients with urticaria or skin disorders who are infected by the parasite [24]. In the present study, similar to other reports [7, 25, 26] *Blastocystis* was the most prevalent parasite in the studied population, yet there was no relationship to abnormal stool consistency. However, in our previous study from Isfahan, the infection rate was significantly higher in patients with loose stool [25]. Additionally, in the present study, *Blastocystis* was more prevalent in winter than in the other seasons.

*Endolimax nan*a is considered a nonpathogenic and commensal protozoon of the human colon that remains largely unexplored in terms of morphology, genetic diversity, taxonomy, host specificity, and epidemiology [27]. However, *E. nana* is rarely reported to be responsible for cases of diarrhea [27, 28], abdominal pain, polyarthritis [27], and urticaria [29, 30] in some individuals. Some reports considered *Blastocystis* and *E. nana* to be associated with diarrhea in children when they occur at a high prevalence and intensity [28]. In the present study, *E. nana* infection was ranked second in terms of prevalence, yet there was no association with diarrhea. The prevalence was significantly high during winter.

*Entamoeba coli* is also considered a nonpathogenic organism of the gastrointestinal tract of humans [31]. In 1991, *E. coli* was reported in 10 patients with diarrhea, which was resolved after treating the *E. coli* infection [32]. In the present study, *E. coli* infection was in the third rank in terms of prevalence, yet there was no association with diarrhea.

Giardiasis is caused by *G. lamblia* which is an intestinal protozoan flagellate that infects the upper small intestine and causes acute watery diarrhea, yet some infections may be asymptomatic. It is suggested that infection with *Giardia* can be protective against other diarrheal diseases. *G. lamblia* may also result in irritable bowel syndrome (IBS) and food allergies after clearance [33]. In the present study, giardiasis was the fourth most prevalent protozoan (0.5%), prevalent in summer and winter. The observed prevalence is either close to some other recent reports from Iran, for instance, Shiraz [7], or lower than the report from Roudehen in Tehran Province [34].

*Cryptosporidium* is a protozoan that is well known for its resistance to chlorination during water treatment. It is transmitted by the fecal-oral route through contaminated food or water, man-to-man or animal-to-man contact, and environmental exposure and resides in the small intestine of hosts. Acute gastrointestinal symptoms are usually self-limiting, but they could be problematic in immune deficiencies [35]. In the present study, all cases with cryptosporidiosis had loose (50%) or watery stool (50%), and no coinfection was observed with *Cryptosporidium*. The observed prevalence in Urmia is considerably lower than most of the previous reports from different regions of the country [36, 37] and Urmia [20, 38].

*Enterobius vermicularis*, or pinworm, is among the most prevalent nematode infections worldwide. Humans are the only natural host for *E. vermicularis*, and it is spread among people living in crowded environments directly through egg ingestion. The diagnosis of enterobiasis can be achieved by cellophane tape or pinworm paddle tests, in which adhesive tape is placed on the perianal area and then examined under a microscope to detect eggs. Stool examination is not recommended for the diagnosis of enterobiasis, yet eggs are rarely detectable in stool [39]. In the present study, an accidental case of enterobiasis was diagnosed by observation of eggs in the stool examination.

Salehi et al. (2020) carried out research on IPIs in 52 children with cancer in Ahvaz, Southwest Iran. They reported that 38.38% of their studied population was infected/colonized by a variety of intestinal parasites/commensals. Similar to the present study they reported *Blastocystis* (23%), *Endolimax nana* (7.7%), *Entamoeba coli* (1.92%), and *Chilomastix mesnili* (1.92%) as the most prevalent protozoa. However, they also reported *Strongyloides stercoralis* (3.84%) which was not detected in the present study. They concluded that *Blastocystis* and *Endolimax* nana are the most prevalent in individuals admitted to Baqaei2 Hospital of Ahvaz, Iran [26]. Comparing their reported prevalence with the present study shows that IPIs/Cs in Ahvaz in children with cancer are considerably higher than our results in Urmia.

Abbaszadeh et al. (2021) in a meta-analysis studied intestinal parasites among intellectually disabled individuals in Iran. They reported an overall pooled prevalence of 41% with a range of 21–68% across subgroups. The most prevalent reported parasites were *Entamoeba coli* (16.2%), *Blastocystis* spp. (12.2%), *Giardia duodenalis* (11.9%), and *Enterobius vermicularis* (11.3%) followed by *Strongyloides stercoralis* (10.9%) and *Hymenolepis nana* (2.8%) [40]. Based on the results of their study, IPIs are highly prevalent in intellectually disabled humans compared to our studied population (6.5%).

Teimouri et al. (2020) studied the prevalence of intestinal parasites among food handlers in Iran in a meta-analysis. Their findings showed the overall pooled prevalence of IPs as 19.3%. They reported a significantly higher prevalence of protozoan parasites (20%) compared to helminthic parasites (1.6%). The reported parasites were *Giardia lamblia* (5.2%), *Entamoeba coli* (5.0%), *Blastocystis* spp. (4.4%), *Ascaris lumbricoides* (1.4%), *Enterobius vermicularis* (0.9%), and *Hymenolepis nana* (0.5%). Additionally, food handlers with lower education were 20% more exposed to IPIs [41]. Based on their report, the prevalence of IPIs among food handlers in Iran is considerably high, approximately three times higher than that in our study population, which is a risk factor for spreading directly transmitted parasites in society.

## Conclusion

According to the results, the most prevalent IPIs in West Azerbaijan Province are caused by *Blastocystis* spp., and *Giardia lamblia*; however, most intestinal protozoa observed in the study were nonpathogenic and commensal, which shows water or food contamination in the area. In terms of helminthic infections, no helminths were detected except for one accidental case of enterobiasis; thus, West Azerbaijan is in a decent condition compared to past studies in Urmia and other regions of the country. Furthermore, medical technologists in the parasitology section of medical laboratories and physicians must be trained and aware of the presence of intestinal parasites.

## Data Availability

Results data (raw or analyzed) will be available and presented by contacting the corresponding author.
